# Recommended long term care settings following aged care assessments in Australia

**DOI:** 10.1371/journal.pone.0204342

**Published:** 2018-11-29

**Authors:** Marijan Jukic, Jeromey B. Temple

**Affiliations:** Demography and Ageing Unit, Melbourne School of Population and Global Health, University of Melbourne, Melbourne, Victoria, Australia; University of West London, UNITED KINGDOM

## Abstract

The purpose of this study was to examine the prevalence and correlates of recommended long term care settings following aged care assessments in Australia. Using unique administrative data on 500,000 aged care assessments, we utilized multinomial logistic regression models to estimate the association between characteristics of the individual (their assistance needs, health conditions and demographic characteristics) and the recommended long-term care setting. The vast majority (94%) of recommended long-term care settings were for private residences (54%) or residential care (40%). Persons assessed in a setting other than a private residence were unlikely to have a recommended setting for a private residence. Consistent with the assessors toolkit, assistance needs were strongly associated with long term care recommendations. Results provide strong support for the evidence-based approach of aged care assessments in Australia. Nonetheless, with improvements in administrative data linkages and ongoing policy reforms, further analysis is required to reinforce extant policy guidelines.

## Introduction

Between 2015 and mid-century, the proportion of the Australian population aged 65 years and over is projected to increase from under 15% to 21% [1]. In June 2015 approximately 176,967 people lived in residential care, with about 96% being aged 65 and over [2]. However, under 5% of all persons aged over 65 were in care at this time, although lifetime prevalence of usage is higher [3]. This is consistent with the broader Australian policy of ageing in place, encouraging independent living in the community through community care services as strengthened in the Aged Care (*Living Longer Living Better)* Bill 2013. Although low proportions of those aged over 65 currently live in care, numerically the number of older Australians living in residential care is projected to increase substantially due to population ageing [4].

In Australia, movement from the community into residential care is governed by the Aged Care Assessment Program (ACAP) which operates under the *Aged Care Act 1997*. Under this Act, Aged Care Assessment Teams (ACAT) make recommendations about individuals’ care setting transitioning into residential care from the general community. The recommended long-term care setting (RLTC) can include living in the community (e.g. private residence, independent living), in residential care, hospitals or other types of institutional care. The recommendations made as part of this assessment are independent of the individuals’ approval to access residential care. Moreover, ultimate approval and placement in residential care is affected by a range of factors including supply of places in local areas, a means and needs test in addition to the residential care facilities approval of the client. However, the aged care assessment, known as an ACAP assessment, is an important key first step in entry into residential care in Australia. It is also a compulsory step in gaining access to a range of support services including home care, respite care and flexible care packages. These services are provided in a setting other than residential care, such as the clients family home, hospitals or independent living facilities.

Understanding movement through the aged care system is important for Federal and State government planning and financing purposes. Indeed, several key studies have detailed the use of aged care services prior to death and the role of disease in explaining entry to care and use of services [5–6]. Considerable descriptive analysis has also been produced by the Australian Institute of Health and Welfare on pathways between hospitals and residential care, use of aged care service by different demographic groups and general detailed statistical overviews of aged care in Australia [6–10]. However, more detailed multivariate studies of aspects of aged care assessments, approvals and subsequent entry to care are rare in Australia. A notable exception, is the recent work examining the correlates of entry to residential care [11–12].

Kendig et al [11–12] and Wang et al [13], in their Australian studies, confirm that a range of functional, mental and physical health conditions is closely associated with entry to and probability of being in residential aged care, consistent with findings from the US, Netherlands and Canada [14–20]. Indeed, assistance needs, together with age, are the most important group of factors that determine an individual’s entry to residential aged care [18,21]. This is consistent with earlier findings that age, functional status and mental status are the best predictors of nursing-home entry [22–24].

However, an important gap in our understanding about entry to residential care in Australia, is that there have been no studies quantifying and modelling the detailed recommendations made about the individual care settings during the ACAP process. That is, there is a lack of understanding on how ACATs recommend differential care settings for older Australians, whether in the community or in institutions. In this paper, we address this research gap through answering three questions. First, what is this distribution of recommended long-term care settings (RLTC) made by ACAT teams in Australia (e.g. private residence, residential care). Second, how do these recommendations differ by the clients’ demographic, and health characteristics? Finally, how important are assistance needs (e.g. assistance with self-care) and carer availability in the ACATs recommendations?

### Aged care assessment program background

There is considerable heterogeneity in the systems for care in the community and in residential care among OECD countries. Australia is relatively unique in that it has a nationally standardised assessment tool, administered during the ACAP assessment, which is used to determine access to government funded services in and out of the community. Broadly, Australia can be viewed as a mix of liberal systems (like the UK—oriented towards tax-based, means-tested and service-oriented support), but complemented by traditional socio-democratic ideas of universal support [25]. As is the case in many countries, except for the USA, Australia has a universal element to social protection for residential care needs [26]. It means that residential care homes receive full Government assistance in cases where aspiring residents are assessed to be below the 'minimum permissible asset value', but these RLTC settings are not obliged to accept applications, either because of a lack of available places or inability to provide the required care services [27].

The objective of the ACAP program is to assess care needs and assist frail clients to access appropriate care and by doing so, improve the health and wellbeing of older Australians [28]. An ACAP assessment is done when ACATs receive requests or referrals for assessment of an aged person from any potential source. An ACAT recommendation is required for specific Commonwealth subsidised aged care services including residential aged care, home care or flexible care in the form of transition care. The recommendation made by the ACAT is the first compulsory step in obtaining access to these care services, although it does not guarantee ultimate admission to a care facility.

**Fig 1:**
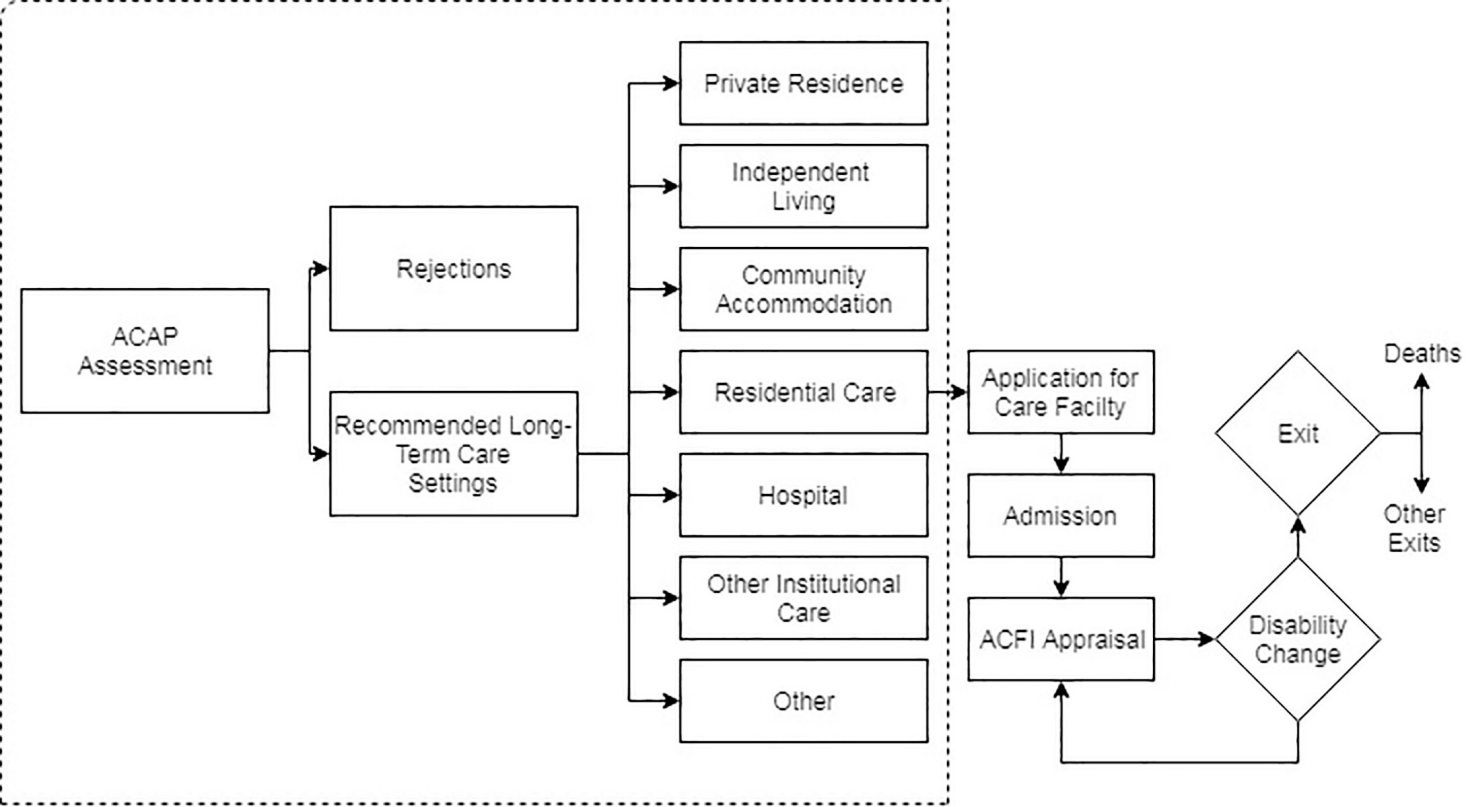
Individuals idealised pathway to care in Australia. Note: Our analysis pertains to the ACAP assessment in the highlighted box.

The *Aged Care Act 1997* requires that an ACAP assessment is undertaken by registered ACATs, who make a recommendation about the optimal living and care arrangements of the client, referred to as a long-term care setting recommendation [29]. Fig 1 displays a typology of an individual’s movement following an ACAP assessment. At the point of assessment, an individual’s ACAP can be approved for care in a RLTC setting or unapproved. A person is eligible for approval for care if they meet the eligibility requirements under division 21 of the Act and Part 2 of the Approval of Care Recipient Principles 2014. Where a person is determined as not meeting the eligibility requirements of any care programme under the Act, the assessment would result in “No Care Approved”.

Not all assessments result in an approved permanent residential care place, requiring alternative RLTC settings. These RLTCs include private residence, independent living, community accommodation, residential care, hospital, other institutional care or other arrangements. As noted earlier, services provided in these settings can include home care, respite care, transition care or short-term restorative care.

For those who are recommended to residential care, approval is granted before applications are made to the facility and admission occurs. This component of the pathway is complicated considerably by whether a place is available in the location, and by a means tests, in addition to government budgetary constraints impacting supply. After aged care packages (residential or home and community) are recommended to older persons by ACATs, in many cases there may be a waiting period before actual commencement, and some may not take up an aged care program at all. One reason for this is a lack of availability of appropriate services in specific regions, which is constrained by the allocation of licences from the Government. Indeed, some regions have excess supply and others excess demand for a range of services [30]. Both providers and consumers have expressed concerns pertaining to the allocative efficiency specifically in this component of the pathway [31–32].

At the point of admission, an ACFI (Aged Care Funding Instrument) appraisal is conducted to assess clients’ assistance and care requirements across three domains: daily living, behavioural needs and complex health care (Fig 1). The ACFI appraisal is updated as clients’ needs change and the subsequent subsidy paid by government to the facility is adjusted.

Our paper focuses specifically on the assessors’ recommendation as part of the ACAP process (highlighted box in Fig 1). The definitions of the RLTCs are given in Table 1 [33].

**Table 1:** The definitions of the recommended long-term care settings.

RLTCs	Description
Private Residence	Houses, flats, units, caravans, mobile homes, boats and marinas
Independent living within a retirement village	Living in self-care independent living units, regardless of whether it is owned or rented. Where provision of care is provided in this setting, it is coded as ‘supported community accommodation’
Supported community accommodation	Living settings or facilities in which support or care is provided by staff or volunteers. Examples include domestic scale-living facilities (e.g., group homes for people with disabilities, congregate care apartments), large scale supported care facilities
Residential care	Residential aged care services, multipurpose services and multipurpose centres
Hospital	Long-term care in a hospital setting
Other institutional care	Other institutional settings providing care (e.g. Hospices, long stay psychiatric institutions)
Other	All other types of community settings

The decision on RLTC settings is based on the physical, psychological, medical, cultural, social and restorative care needs of the client. The ACAT also considers the client’s usual accommodation arrangement, financial circumstances, need for assistance, and access to transport and community support systems. The needs of the carer and the client’s personal choice are also considered.

As part of the initial assessment and needs identification, the ACAP toolkit for assessors provides a set of appropriately validated tools that are nationally consistent. From late 2015, assessors began using assessments through the My Aged Care online portal using the National Screening and Assessment Form. After the initial assessment, the ACAT develops a care plan detailing the service needs of the client. The ACATs then engage in ‘care coordination to the point of effective referral’ [28]. This includes supporting the client and their support network in implementing the care plan and accessing services.

## Data and methods

### Data

For this study, we utilise unique unit record administrative data on over 800,000 aged care assessments from the National Aged Care Data Clearing House (NACDCH) [34]. The ACAP data are derived from ACAT assessments with more than 150,000 assessments completed each year. Upon completion of the assessment, the recommendations of the ACAT team are ultimately provided to the NACDC.

The confidentialised ACAP file includes all ACAP referrals received by ACAT members from 1 July 2010 to 30 June 2013. Prior to our analyses, we removed 236,595 records where the assessment was not complete. A further 97,807 records were found to be duplicates, with the same approval and referral dates. An additional 2,101 records were incomplete (with missing assessment date) and 49 cases where the gender of the client was unknown and removed. Finally, 18,484 cases for persons aged under 65 years were deleted. Removing these duplicate, incomplete and out of scope records, yielded a final sample of 492,628 ACAP assessments for those aged 65 years and over. We limit our analysis to those aged 65 and over as this population is not eligible for services through the National Disability Insurance Scheme (NDIS).

To maintain client confidentiality, not all variables from the ACAP assessment are made available to researchers. One particularly important omission is geocoded records. Supply constraints are an important factor in the allocation of residential care and home care services. Ideally, access to data at Aged Care Planning Region level or lower would enable detailed analyses of the relationship between ACAPs, allocation, unmet demand and supply differences. Nonetheless, these data provide important measures of the characteristics of the individual and their care including age, carer availability, health conditions, sex and state of residence and RLTC setting.

### Approval to use data

These data were made available to the authors by the AIHW as part of ongoing improvements in access to aged care data. The data were collected by the Australian Federal Government under the *Aged Care Act* and a de-identified data set is provided to registered users for research purposes.

### Measurement

A number of measures of the health of ACAP clients are available. Clients’ health conditions are grouped into 18 categories using the International Classification of Diseases (ICD) framework [35]. As well as analysing RLTC settings by ICD type, we also analyse differences by counts of health condition types, as a measure of comorbidities.

In addition to detailed measures of the health of clients, the confidentialised data file includes a number of measures of Activities of Daily Living (ADL). These variables provide important measurement of the functional status of older Australians, with specific reference to their needs for assistance with communication, moving, walking, self care, transport, meals, domestic, social and health activities (Table 2) [33].

**Table 2:** ADL assistance needs definition.

ADL	ADL Description
	*Needing the assistance or supervision of another person with*:
Move	Activities such as maintaining or changing body position, carrying, moving and manipulation
	objects, getting in or out of bed or a chair
Moving	Walking and related activities, either away from home or away from home
Communication	Understanding others, making oneself understood by others
Health	Taking medication or administering injections, dressing wounds, using medical machinery,
	manipulating muscles or limbs, taking care of feet (includes a need for home nursing and allied
	health care, such as physiotherapy and podiatry)
Self Care	Daily self-care tasks such as eating, showering/bathing, dressing, toileting and managing
	incontinence
Transport	Using public transport, getting to and from places away from home or driving
Social	Shopping, banking, participating in recreational, cultural or religious activities, attending
	day centres, managing finances and writing letters
Domestic	Household chores such as washing, ironing, cleaning and formal linen services
Meals	Meals, including the delivery of prepared meals, help with meal preparation and managing
	basic nutrition
Home	Home maintenance and gardening
Other	Needs assistance with other tasks not stated above

The measure of the clients living arrangement collected by the ACAT assessor is their usual accommodation setting. If the ACAP assessment is conducted away from the usual accommodation setting and is temporary in nature, the assessor records the usual setting, not the place of the ACAP assessment. For example, if a person who usually resides in a private residence is assessed while temporarily in hospital, the usual accommodation setting is coded as private residence.

### Statistical model

To examine the association between demographic characteristics, assistance needs, health characteristics andrecommended long term care setting, we utilized Stata 14.0 [36]. As the dependent variable RLTC is polytomous (6 categories) we specified a multinomial logistic regression model (MNL). Using the raw MNL coefficients, we generate Relative Risk Ratios (RRR) which measure the change in the odds of observing a given RLTC setting, given a one unit change in each of our demographic, health and assistance needs measures. A RLTC setting in a private residence is the base group for the MNL specification. In this way, the interpretation of the RRR is analogous to the use of odds ratios in standard logistic regression.

We tested the stability of our parameter coefficients by splitting the sample into each RLTC pair groups (e.g. private residence v independent living or private residence v residential care) and estimated standard logistic regression models. We also compared these coefficients with those obtained from skewed logistic regression and penalized maximum likelihood estimation [37–38]. Comparisons between the models show very strong consistency in the direction and significance of coefficients. Herein, we present the MNL model for simplicity.

To examine the relative size effects of demographic, health and assistance needs on accessors recommendations, we present several measures of pseudo r2 statistics as well as information criterion measures. Specifically, we present McFadden, Cox-Snell and Cragg-Uhler pseudo r2 measures which indicate improved model fit with higher r2 estimates. Of the information criterion statistics, we include both AIC and BIC tests. We follow Raftery’s [39] procedure to assess improvements to model fit. For a detailed discussion of each of these measures, see Long [40].

Because some individuals have multiple ACAP assessments, it is important to adjust for potential intragroup correlation. We produce cluster-robust variance estimates to ensure corrected standard errors and variance-covariance matrix of the estimators. Standard post estimation testing is used to ensure the veracity of the underlying statistical models. Initially, we estimate two types of models: one using counts of the number of types of health conditions and number of ADLs and a second model including individual ADL and health conditions. These variables cannot be included in the same model due to multicollinearity biasing the parameter estimates. We conduct model selection tests and find very strong support for the individual conditions and individual ADL models [39]. Nonetheless, we also report parameter coefficients for the separate models using count variables in the text to contextualise the detailed regression results. After model fitting, we conduct condition tests to ensure absence of multicollinearity due to the inclusion of ICD and ADL types in the same model. The resulting condition numbers were very low, supporting the underlying model specification.

## Results

Of the 492,398 completed ACAP assessments between 2010 and 2013 for those 65 and over, the vast majority of recommendations are for either a long term care setting in private residences (54%) or residential care (40%) (Table 3). A further 4% of cases involve recommendations for independent living, and less than 1% are recommended to community accommodation (0.6%), hospitals (0.1%), other institutional care (0.01%) or other (0.9%). Although the proportions for those outside of private residences and residential care are small, they represent a substantial number of people aged 65 and over. For example, approximately 20,412 recommendations to independent living and almost 3,000 recommendations to community accommodation.

**Table 3:** Current living arrangement by recommended long term care setting (%), 2010–2013.

	Current Living Arrangement^1^								
	Private	Independent	Community	Resi	Hospital	Other	Other	Total^2^	
	Residence	Living	Accom.	Care		Institut.	Care		
	%	%	%	%	%	%	%	%	n
**Recommended Long Term Care (RLTC) Setting**									
Private Residence	*61*.*5*	20.0	6.3	1.3	7.0	2.5	31.9	54.0	265,963
Independent Living	0.5	*35*.*8*	0.7	0.2	1.2	0.0	1.0	4.1	20,412
Community Accomodation	0.2	0.2	*28*.*0*	0.0	0.8	2.2	2.6	0.6	2,714
Residential Care	36.9	43.1	63.7	*97*.*9*	86.8	86.0	56.0	40.2	198,129
Hospital	0.0	0.0	0.0	0.0	1.4	0.0	0.1	0.0	239
Other Institutional Care	0.0	0.0	0.1	0.0	0.8	*8*.*4*	0.1	0.0	179
Other	0.8	0.7	1.1	0.3	0.8	0.8	*8*.*2*	0.9	4,294
Total	100	100	100	100	100	100	100	100	
n =	419264	51624	6619	17000	552	418	5842		492,398
%	85.1	10.5	1.3	3.5	0.1	0.1	1.2		100

Notes:

^1^ The measure of the clients living arrangement collected by the ACAT assessor is their usual accommodation setting. If the ACAP assessment is conducted away from the usual accommodation setting and is temporary in nature, the assessor records the usual setting, not the place of the ACAP assessment. For example, if a person who usually resides in a private residence is assessed while temporarily in hospital, the usual accommodation setting is coded as private residence.

^2^ Includes 468 cases where current living arrangement is unknown.

Interestingly, there are strong differences in RLTC setting by the client’s usual current living arrangement. For example, about 60% of those currently in a private residence have a RLTC setting of private residence and a further 37% have a residential care RLTC setting. For those currently in residential care, about 98% have a RLTC to remain in a residential care setting. High proportions of those currently in hospital also have a residential care RLTC setting (87%) as do those currently in other institutional care (86%).

Throughout the population aged 65 and over, the bivariate comparisons show important differences in the demographic characteristics of those in alternative RLTC settings (Table 4). Relative to those recommended to a private residence, those in independent living or other care are more likely to be female. Older people are more likely to be recommended a care setting in any non-private residence setting with the exception of other institutional care. We also observe strong differences in the RLTC setting by cultural identity. Indigenous Australians are over represented in community accommodation and those born overseas are less likely to be in independent living, community accommodation or residential care when compared to living in a private residence. Not surprisingly, having a co-resident carer or co-resident family member is strongly associated with having a RLTC setting in a private residence.

**Table 4:** Demographic, health and need (ADL) characteristics by recommended long term care setting (%), 2010–2013.

	Recommended Long Term Care (RLTC) Setting														
	Private		Independent		Community		Resi		Hospital		Other		Other		Total
	Residence		Living		Accom.		Care				Institut.		Care		
	%		%		%		%		%		%		%		%
*Sex*															
Male	38.5	-	31.6	***	38.4		39.5	***	49.0	**	46.9	*	36.3	**	38.6
Female	61.5	-	68.4	***	61.6		60.5	***	51.0	**	53.1	*	63.7	**	61.4
*Age*															
65–74	15.6	-	7.8	***	20.9	***	11.5	***	22.6	**	31.3	***	15.2		13.7
75–84	44.6	-	40.0	***	33.6	***	37.7	***	45.2		38.5		37.7	***	41.5
85+	39.8	-	52.2	***	45.5	***	50.8	***	32.2		30.2	**	47.1	***	44.9
*Cultural Identity*															
Australian Born—Non Indigenous	64.5	-	74.6	***	68.5	***	69.1	***	65.3		68.7		65.3		66.8
Australian Born—Indigenous	0.9	-	0.3	***	5.0	***	0.8	***	0.4		1.1		0.9		0.9
Born Overseas	34.6	-	25.1	***	26.5	***	30.1	***	34.3		30.2		33.9		32.3
*Lives in the Community*															
No	29.1	-	93.6	***	90.6	***	47.1	***	40.6	***	55.9	***	39.2	***	39.5
Yes	70.9	-	6.4	***	9.4	***	52.9	***	59.4	***	44.1	***	60.8	***	60.5
*Family in the Household*															
No	42.2	-	64.6	***	67.6	***	56.2	***	54.8	***	52.0	**	57.2	***	49.1
Yes	57.8	-	35.4	***	32.4	***	43.8	***	45.2	***	48.0	**	42.8	***	50.9
*Carer*															
No	14.7	-	21.5	***	32.1	***	22.0	***	23.8	***	38.0	***	18.4	***	18.1
Carer Coresident	48.3	-	26.6	***	16.2	***	37.2	***	40.2	*	38.0	**	35.1	***	42.6
Carer Non-coresident	37.0	-	51.8	***	51.7	***	40.8	***	36.0		24.0	***	46.5	***	39.3
Health Conditions															
No ICD Conditions reported	0.2	-	0.1	***	0.3		0.1	***	1.7		0.6		0.4		0.2
Circulatory system diseases	78.1	-	78.9	**	73.0	***	79.2	***	64.4	***	69.8	*	82.4	***	78.5
Abnormal laboratory findings	64.0	-	62.5	***	63.0		72.0	***	69.5		57.5		66.8	***	67.2
Muscosceletal system	60.4	-	65.8	***	58.3	*	54.7	***	38.5	***	48.0	***	55.9	***	58.2
Mental & behavioural disorders	38.4	-	36.1	***	46.4	***	51.4	***	39.3		51.4	***	39.5		43.6
Endocrine, nutritional & metabolic	43.7	-	41.0	***	40.6	**	40.9	***	37.2	*	43.6		44.2		42.4
Digestive system diseases	28.8	-	32.3	***	29.1		27.8	***	21.8	**	31.3		29.3		28.5
Genitourinary diseases	25.6	-	24.4	***	24.7		31.2	***	24.3		25.1		32.0	***	27.8
Eye & adnexa diseases	27.1	-	31.1	***	27.5		25.9	***	16.3	***	15.1	***	20.6	***	26.7
Respiratory system diseases	22.9	-	21.5	***	22.1		23.2	*	18.8		23.5		25.4	***	22.9
Neoplasms	20.2	-	20.5		17.6	***	23.4	***	37.2	***	30.2	**	21.6	*	21.5
Congenital malformations	21.4	-	23.4	***	20.3		19.4	***	24.3		21.2		38.2	***	20.8
Ear & mastoid diseases	19.5	-	22.0	***	19.7		20.5	***	10.9	***	14.5		15.3	***	19.9
Nervous system diseases	18.1	-	19.0	**	17.0		18.0		12.1	**	18.4		17.9		18.1
Skin & subcutaneous diseases	7.2	-	7.2		7.1		8.2	***	8.4		5.0		9.5	***	7.6
Blood diseases	6.6	-	6.0	***	6.4		7.3	***	9.6		10.1		9.2	***	6.9
Not elsewhere specified	2.1	-	2.3	*	2.4		2.3	***	2.5		5.0		3.0	***	2.2
Infections & parasitic diseases	1.4	-	1.4		1.0	*	1.7	***	1.7		1.1		5.2	***	1.5
Perinatal conditions	0.2	-	0.3		0.3		0.2	**	0.4		1.7		0.2		0.2
Mean Health Conditions	4.9	-	5.0	***	4.8	**	5.1	***	4.4	***	4.7		5.2	***	5.0
Assistance Needs															
ADL Move	19.2	-	13.4	***	15.6	***	41.8	***	59.4	***	50.3	***	53.8	***	28.4
ADL Moving	54.0	-	47.1	***	48.6	***	73.4	***	79.5	***	73.2	***	79.0	***	61.7
ADL Communication	13.8	-	8.1	***	14.8		27.1	***	28.9	***	31.8	***	21.4	***	19.0
ADL Health	76.4	-	72.5	***	83.2	***	91.1	***	90.4	***	85.5	***	91.9	***	82.3
ADL Self Care	63.8		54.2	***	65.7	*	86.5	***	87.0	***	82.7	***	87.2	***	72.7
ADL Transport	88.3	-	87.6	**	88.4		96.2	***	91.2		91.1		94.0	***	91.4
ADL Social	84.3	-	82.1	***	86.4	**	93.5	***	87.4		88.3		90.3	***	88.0
ADL Domestic	95.4	-	95.8	***	93.7	***	87.2	***	85.4	***	72.6	***	93.5	***	92.0
ADL Meals	83.2	-	81.7	***	90.6	***	84.5	***	83.3		69.3	***	91.1	***	83.7
ADL Home	81.1	-	77.4	***	69.5	***	72.8	***	66.9	***	57.5	***	79.0	***	77.4
ADL Other	5.3	-	5.2		5.7		5.8	***	3.3		2.8	*	2.6	***	5.5
Mean ADL	5.8	-	5.4	***	5.7		6.8	***	6.8	***	6.4	***	6.9	***	6.2
Total	266002		20417		2714		198179		239		179		4295		
Distribution (%)	54.0		4.1		0.6		40.2		0.0		0.0		0.9		100.0

Notes:—comparison case for tests of proportions

*** p<0.001

**p<0.01

*p<0.05; RAC Residential Aged Care

In addition to demographic factors, the RLTC setting differs strongly by health conditions and ADL assistance needs. Particularly strong differences in RLTC settings are observed for mental and behavioural disorders, which includes conditions such as dementia and schizophrenia. This is consistent with previous Australian and international studies [41–42]. For example, 38% of those with a private residence RLTC have this condition type, compared with over 50% of those with a residential care or other institutional care RLTC setting. High proportions of neoplasms are found for those with a hospital (37%) or other institutional care (30%) setting. Within the full population, there is a high presence of cases with circulatory system diseases (82%), abnormal laboratory findings (67%) and musculoskeletal system and connective tissue diseases (56%).

Supporting these differences by ICD type, there are considerable variations in RLTC setting by ADL assistance needs. With the exception of ADLs for home maintenance, those with a residential care RLTC setting have much higher ADL needs across all categories compared to those in private residences. For example, 42% of those with a residential care RLTC setting have an ADL assistance need for body movements compared with under 20% of those in a private residence setting.

Although these bivariate comparisons show clear differences in the RLTC settings made by differing demographic and health characteristics, the magnitude of differences may be masked by confounding factors. For example, are many ADL differences so strong because of higher needs with increasing age? To isolate these effects, we estimated a multinomial logit model with a setting in private residence as the comparison group (Table 5). For example, the first column reports on the determinants of receiving a recommendation to an independent living RLTC versus private residence. Column 2 reports the relative risk ratios for community accommodation versus private accommodation and so on. The relative risk ratios presented here show the association between each variable and the odds of belonging to a RLTC other than private residence.

**Table 5:** MNL regression model of recommended long term care settings.

	Comparison = Private Residence											
												
	Independent		Community		Resi		Hospital		Other		Other	
	Living		Accom.		Care				Institut.			
	RRR		RRR		RRR		RRR		RRR		RRR	
Demographic Factors												
Male	1.00		1.00		1.00		1.00		1.00		1.00	
Female	0.98		0.85	***	0.86	***	0.76	+	0.97		1.04	
65–74	1.00		1.00		1.00		1.00		1.00		1.00	
75–84	2.25	***	0.89	*	1.36	***	0.60	**	1.00		1.31	+
85+	3.05	***	1.35	***	1.95	***	0.57	**	1.31	***	2.15	***
Non Indigenous	1.00		1.00		1.00		1.00		1.00		1.00	
Indigenous	0.18	***	2.80	***	0.75	***	0.61		0.90		0.74	
Born Overseas	0.61	***	0.70	***	0.76	***	0.73	+	0.94	+	0.70	***
Lives in Community—No	1.00		1.00		1.00		1.00		1.00		1.00	
Yes	0.03	***	0.04	***	0.52	***	0.46	***	0.67	***	0.45	***
*Family/Others in the Household—No*	1.00		1.00		1.00		1.00		1.00		1.00	
Yes	0.74	***	1.22	***	0.73	***	1.05		0.73	***	0.52	***
Carer—No	1.00		1.00		1.00		1.00		1.00		1.00	
Carer Coresident	0.57	***	0.11	***	0.48	***	0.33	***	0.46	***	1.64	**
Carer Non-coresident	1.06	*	0.73	***	0.80	***	0.50	***	0.96		1.05	
Health Conditions												
Infections & parasitic diseases	1.08		0.67	+	1.10	***	0.63		3.07	***	0.48	
Neoplasms	1.08	***	0.91	+	1.26	***	1.71	**	1.11	***	0.27	***
Blood diseases	0.89	***	0.98		1.06	***	1.59	+	1.27	***	0.67	
Endocrine, nutritional & metabolic	0.93	***	0.89	**	0.96	***	1.07		1.05		0.57	***
Mental & behavioural disorders	1.09	***	1.40	***	1.70	***	1.52	**	1.19	***	0.40	***
Nervous system diseases	1.27	***	1.05		0.98	*	0.88		0.97		0.53	**
Eye & adnexa diseases	1.03		0.98		0.92	***	0.58	*	0.76	***	0.44	***
Ear & mastoid diseases	1.01		0.96		1.00		0.94		0.83	***	0.75	
Circulatory system diseases	0.98		0.79	***	1.02	*	0.74	+	1.20	***	0.28	***
Respiratory system diseases	0.85	***	0.85	**	1.02	*	0.99		1.11	**	0.42	***
Digestive system diseases	1.07	***	0.98		0.97	***	1.22		1.02		0.64	**
Skin & subcutaneous diseases	0.96		0.93		1.01		0.66		1.12	*	0.64	
Muscosceletal& connective tissue	1.06	***	0.93		0.83	***	0.80		0.85	***	0.32	***
Genitourinary diseases	0.98		0.99		1.13	***	0.91		1.16	***	0.68	*
Perinatal conditions	1.27		1.34		0.90		5.83	**	0.84		-	
Congenital malformations	1.12	****	0.91	+	0.73	***	0.87		1.69	***	0.35	***
Abnormal laboratory findings	0.95	**	0.98		1.26	***	0.75	+	0.99		0.42	***
Not elsewhere specified	1.23	***	1.16		1.10	***	2.15	*	1.42	***	0.30	*
Assistance Needs												
ADL Move	0.92	***	0.94		2.16	***	3.03	***	3.28	***	1.32	
ADL Moving	0.89	***	0.80	***	1.27	***	1.38	+	1.56	***	1.11	
ADL Communication	0.71	***	1.20	**	1.54	***	1.94	***	1.29	***	1.15	
ADL Health	1.01		1.64	***	1.60	***	1.06		1.90	***	0.65	***
ADL Self Care	0.78	***	1.18	***	2.14	***	1.65	*	1.73	***	0.96	
ADL Transport	0.97		0.83	**	1.50	***	1.11		0.82	**	0.48	***
ADL Social	0.84	***	1.08		1.52	***	1.39		1.04		0.74	**
ADL Domestic	1.70	***	0.75	**	0.31	***	0.24	***	0.45	***	0.14	***
ADL Meals	1.18	***	3.82	***	1.58	***	1.15		2.18	***	1.20	+
ADL Home	1.00		0.65	***	0.77	***	0.60	**	0.88	**	0.44	***
ADL Other	1.01		1.13		1.12	***	0.63		0.49	***	3.23	***
Constant	0.14	***	0.04	***	0.32	***	0.01	***	0.01	***	0.51	***

Notes:

*** p<0.001

**p<0.01

*p<0.05 +p<0.10; RRR Relative Risk Ratio–adjusted for all model covariates; Resi Care—Residential Care

Relative Risk Ratios measuring the contribution of demographic factors strongly support the bivariate results, once all other variables are controlled for. With increasing age, individuals are less likely to have a private residence RLTC setting. For example, those 85 and over are over 3 times more likely to have an independent living RLTC relative to private residence (RRR = 3.1, p<0.001). Cultural identity remains strongly associated with RLTC setting. Older Indigenous Australians are about 2.8 times more likely to be in community accommodation (RRR = 2.80 p<0.001) compared to the non-Indigenous Australian born clients. Those born overseas remain more likely to hold a private residence RLTC (eg. residential care RRR = 0.76 p<0.001). Co-residence of a carer or family member also remains strongly associated with RLTC setting. For example, those with a co-resident carer are about 90% less likely to live in community accommodation (RRR = 0.11 p<0.001) and 52% less likely to have residential care RLTC setting (RRR = 0.48 p<0.001) when compared to those with no carer. The variable ‘lives in the community’ is included to control for the current setting of the individual, independent of the RLTC setting and shows that those currently living in the community are at a far lower odds of having a different RLTC setting.

Controlling for these important demographic differences, health conditions remain strongly associated with RLTC settings. Specifically, there is considerable variation in which specific disease types are related to alternative RLTC settings. For example, having a hospital RLTC setting is strongly associated with neoplasms (RRR = 1.71 p<0.01), mental and behavioural disorders (RRR = 1.52 p<0.01) and perinatal conditions (RRR = 5.83 p<0.01). Having a RLTC setting in residential care is associated with many more conditions, including higher incidences of infections and parasitic disease (RRR = 1.1 p<0.001), neoplasms (RRR = 1.26 p<0.001), blood disease (RRR = 1.06 p<0.001), mental and behavioural disorders (RRR = 1.7 p<0.001), circulatory and respiratory (RRR = 1.02 p<0.05), genitourinary (RRR = 1.13 p<0.001) and abnormal laboratory findings (RRR = 1.26 p<0.001). As discussed earlier, alternative models were fitted with counts of the number of conditions. Each additional condition was found to add 2% to the probability of having a RLTC setting of residential care (RRR = 1.02 p<0.001). Conversely, each additional condition reduced the probability of having a RLTC in community accommodation (5.6% RRR = 0.94) or other care (RRR = 0.40 p<0.001).

Independent of health conditions, ADL assistance needs are strongly associated with recommended RLTC settings. Having a residential care setting RLTC, in particular, is associated with multiple ADL types. For example, with the exception of domestic and home ADLs, those with a residential care RLTC are far more likely to have all other ADL assistance needs. The associations are particularly strong for self care (RRR = 2.14 p<0.001) and body movements (RRR = 2.16 p<0.001). The lower likelihood for home and domestic ADL needs for residential care reflects the coding of these two variables for those currently living in this setting. Strong associations between ADL needs and RLTC settings are also observed in hospital and other institutional care settings. For example, those with ADL assistance needs with body movements are 3.03 times (p<0.001) more likely to be in hospital RLTC setting and 3.3 times (p<0.001) more likely to be in another institutional care setting relative to a private residence. Not surprisingly, those in an independent living setting have higher ADL needs for domestic assistance and meals, but lower or no different ADL needs on all other measures compared to those in a private residence setting. For the alternative models we fitted, using the number of ADLs as an independent variable, each additional ADL type increased the probability of having a residential care RLTC setting by 1.5 times (RRR = 1.51 p<0.001). The corresponding figures for hospital and other institutional care are 1.32 (p<0.001) and 1.63 (p<0.001) respectively. Not surprisingly, each additional ADL decreases the probability of being in independent living by 7% (RRR = 0.93 p<0.001).

As a summary measure of the relative importance of demographic, assistance needs and health conditions on assessors’ recommendations, we calculate a number of scalar measures of fit (Table 6). Regardless of whether these variables are introduced individually (single models) or additively (additive models), these results indicate the considerable strength of assistance needs (ADLs) in explaining assessors’ recommendations. For example, the baseline demography model (including age, cultural identity, age, carer availability and community residence flags) has an r2 of approximately 0.154 on the Cragg-Uhler measure. This doubles to 0.3169 when ADL factors are added. This considerable improvement to fit is mirrored by a commensurate drop in both AIC and BIC statistics. Following Raftery’s [39] approach, this provides very strong support for a model including ADLs in addition to a model with demographic characteristics only. With the addition of health conditions to the model, the pseudo r2 rise and information criterion measures fall–again consistent with improved fit. Nonetheless, the relative improvement to fit is lower when compared to the addition of ADLs. In summary, these results underscore the importance of ADLs in an assessor’s determination of a clients recommended care setting.

**Table 6:** Measures of fit, demography, assistance needs and conditions.

	Pseudo r2			Information Criterion	
	McFadden	Cox-Snell	Cragg-Uhler	AIC	BIC
***Single Models***					
Demography Only	0.076	0.129	0.154	829003	829670
ADL Only	0.094	0.157	0.187	812830	813630
Conditions Only	0.025	0.045	0.053	874574	875840
***Additive Models***					
Demography	0.076	0.129	0.154	829003	829670
Demography + ADL	0.171	0.267	0.319	744027	745426
Demography + ADL + Conditions	0.185	0.286	0.341	731455	734054

Notes: Single Models–groups of covariates introduced individually. Additive models–groups of covariates added sequentially.

## Discussion

### Key findings

Due to population ageing, increasing demands on residential care and home care services are likely to be significant over the coming decades. Heretofore, a gap in our current understanding about the ACAP process is the decisions made by ACAT teams about RLTC in the community, residential care or other institutional settings. In this paper, we have examined (i.) the distribution of RLTC settings between 2010 and 2013, (ii.) how these recommendations vary by the demographic and health characteristics of the client, and (iii.) the role of the ACATs determination of assistance needs (ADLs) and carer availability on alternative RLTC setting.

The majority of older Australians have a preference to receive care in the community, rather than institutions [43]. Findings here show that just over half of all RLTC settings are in the private residences. For those who currently live in a private residence, the figure is higher (61%). However, we also show the considerable variation in recommended long term care (RLTC) settings made by ACATs by the client’s current living arrangement. Those currently in care, hospital or other institutional care are relatively rarely recommended to return to living in private residences. The majority of those currently in these three categories have a RLTC setting of residential care. Those living in independent living (retirement villages) are the only group where a sizeable minority (20%) are recommended to live back in private residences. This indicates that once a recommended setting is made outside of private residences, the individual is unlikely to be recommended to return to a private residence. This provides some evidence that ACAT teams are on average, making reasonable RLTC setting recommendations.

In addition to understanding the RLTC settings in the context of the ACAP process, living arrangement decisions are important for other reasons. Gerontological studies show living arrangements are key predictors of need and well-being in later life [44–45]. Where close familial support in unavailable, many older Australians require additional resources to fulfil this unmet need, placing greater strain on government services [44]. Results from this analysis of administration data clearly support the role of having a partner or a carer on differential RLTC settings. This is consistent with descriptive findings from a cohort study that shows 80% of cohort members living in the community when an ACAP assessment is conducted had a carer [46]. Having a partner or co-resident carer is strongly associated with living in a private residence, and in particular, strongly negatively associated with being in residential care, hospital or other institutional care. This is important as future projections of Australian mature age living arrangements show a significant growth in the number of older Australian males living alone in the community [4]. This implies that there may be greater demands for institutional and in home care due to the lack of a potential carer in the households in the future.

The association between ADLs and care settings are reflective of the toolkit used by ACAT assessors, which collects information on a broad range of health conditions and assistance needs of clients. We expected, and found, that multiple ICD types were clustered in RLTC settings in residential care. Moreover, although hospitals had fewer associations with multiple condition types, the condition types tended to be more severe, such as neoplasms and perinatal conditions. RLTC settings in independent living tended to have weaker odds ratios, with the exception of some nervous system diseases.

The modelling strategy we adopted allows an examination of the contribution of ADL assistance needs to the care setting, independent of health conditions of the client. Moreover, our detailed scalar fit statistics underscore the primacy of the impact of ADLs on assessors’ recommendations. The findings strengthen our conclusion that ACAT teams consider ADLs as the most important determinants in final RLTC recommendation. Compared to living in private residence, the highest concentration of ADLs, such as assistance with self-care, body movement, moving and health, were found in residential care, followed by hospitals and other institutional settings. Those with an independent living RLTC setting, tended to have low numbers of ADLs–with a focus on assistance needs with just meals and domestic tasks.

The clustering of ICDs and ADLs in residential care, hospitals and other institutional care settings is important given the likelihood of increasing comorbidities and longevity in future cohorts of the aged population [47]. This implies, all other things being equal, that demand for these RLTC settings will be higher, regardless of population increases due to population ageing. In this context, an increased policy focus on co-ordinated care and management of complex conditions within the general community may help reduce stressors on these RLTC settings in the future, specifically upon residential care. Notwithstanding, our data clearly demonstrated that current institutional care clients have high care needs, which require complex clinical care. This type of medical care is usually unsuitable to be delivered in community (i.e. limited access to primary health care services in a timely manner as a significant impediment for optimal public health outcome, especially in rural areas). This indicates that, policy makers should not only consider what types of care may be reasonable delivered in community, but also allow for increased demand for residential aged care beds.

### Future research priorities

Taken together, the patterns and associations between types and implied severity of ADLS, ICDs and RLTC settings provides considerable evidence that many ACAT recommendations are evidence based and consistent with the ACAP toolkit. Our findings suggest several pathways to further examine the determinants of assessors’ recommendations, with a view to improving efficiencies of ACATs recommendations.

Firstly, the administrative data that we use does not link the ACATs recommendations to admissions or entry to residential care or other programs such as flexible care packages. Although some cohort analyses have been conducted on pathways to care by the AIHW [46], further multivariate analysis is required of the drivers of these pathways and the determinants of ACAT recommendations leading to care utilisation. Related to this point, in determining the efficiency of ACAT recommendations, it is important to understand the supply and availability of regional level services. Ideally, data at the level of Aged Care Planning Regions (ACPR) level would be required for this analysis. Unfortunately, geocoded information was not available for this study due to confidentiality concerns. The availability of aged care services, due to factors such as remoteness, might well affect the waiting time before a person is being admitted to residential care or these factors may re-direct an older person towards community care arrangements. Indeed, from an ACAT perspective, assessors are recommended to “note that a care service is available if there are allocated places in the area, regardless of whether there is a vacancy at the time of the assessment and approval of the clients” [28]. That is, geographic availability is considered assessors’ recommendations.

Secondly, analyses of the role of ACAT skill mix and the availability of client medical records on ACAT recommendations would strengthen existing guidelines. Under the policy guidelines, the ACATs should have access to a range of skills and expertise to enable a comprehensive assessment. These can include geriatricians, specialists, GPS, nurses, social workers, physiotherapists, occupational therapists and psychologists. The association between the skill mix of assessors and ultimate recommendations and approvals for care would shed light on how different assessors make differential recommendations. Furthermore, when consent is given, the ACAT can also access information from clients’ medical records from GPs and other health professionals. Analyses of the association between medical file availability and the efficiency of care recommendations would assist policy makers in setting policy guidelines for ACATs. Indeed, international studies shows that detailed medical information improves care placement [48–49].

More generally, findings from this study are also important for understanding future demands on the aged care system in Australia. The differing determinants (demographic, health and assistance needs) of alternative RLTC settings indicate that standard demographic projections with few covariates are unlikely to present accurate estimates of residential care populations and care needs of older Australians. This poses questions for the types of models that may be built to project demand for care in the community and in non-community settings in Australia? Specifically, how can increasing collections of ACAP and other administration data be linked with survey, census and demographic data sources to better understand demands and weakness in service provision in the aged and community care sectors? Moreover, how can government and researchers harness the power of modern econometric and data mining techniques, to better understand pathways of care trajectories?

These data were made available to the authors at a time in which there is much reform in the Australian aged care system, due to many of the longer-term financing and workforce issues also facing long term care in the US [50]. Central to this reform is the introduction of a consumer directed care (CDC) model, with the goal of improving choice and flexibility to consumers. However, the implementation of a CDC model is likely to face obstacles which are particularly apparent in rural areas. Low population density across remote areas already causes aged care facilities to operate on the cusp of viability, organization of care services incurs additional costs (i.e. medical staff who need to travel from cities) and older people have limited access to these services. With future releases of the ACAP administration data, a priority is to examine whether assessors’ recommendations have changed from that observed herein.

Noting these limitations and extensions, results from this study show that about half of all ACAT recommendations are for a care setting in the community, that assistance needs (ADLs) play a strong role in the assessors’ recommendations, and that the clustering of ADL and diseases in residential care and hospital settings lends support to the evidence-based approach of ACAP assessments. With further research into the determinants of ACATs recommendations and the constraints they face, particularly regarding regional level availability of care and services, existing policy guidelines for assessors can be strengthened. This has the potential to contribute to the efficiency of the aged care system in Australia, and by doing so, improve the health and wellbeing of older Australians, their carers and families.
